# Impairment of Sulfite Reductase Decreases Oxidative Stress Tolerance in *Arabidopsis thaliana*

**DOI:** 10.3389/fpls.2016.01843

**Published:** 2016-12-02

**Authors:** Meiping Wang, Yunli Jia, Ziwei Xu, Zongliang Xia

**Affiliations:** College of Life Science, Henan Agricultural UniversityZhengzhou, China

**Keywords:** sulfite reductase, methyl viologen, oxidative stress, glutathione

## Abstract

As an essential enzyme in the sulfate assimilation reductive pathway, sulfite reductase (SiR) plays important roles in diverse metabolic processes such as sulfur homeostasis and cysteine metabolism. However, whether plant *SiR* is involved in oxidative stress response is largely unknown. Here, we show that *SiR* functions in methyl viologen (MV)-induced oxidative stress in *Arabidopsis*. The transcript levels of *SiR* were higher in leaves, immature siliques, and roots and were markedly and rapidly up-regulated by MV exposure. The *SiR* knock-down transgenic lines had about 60% residual transcripts and were more susceptible than wild-type when exposed to oxidative stress. The severe damage phenotypes of the *SiR*-impaired lines were accompanied by increases of hydrogen peroxide (H_2_O_2_), malondialdehyde (MDA), and sulfite accumulations, but less amounts of glutathione (GSH). Interestingly, application of exogenous GSH effectively rescued corresponding MV hypersensitivity in *SiR*-impaired plants. qRT-PCR analysis revealed that there was significantly increased expression of several sulfite metabolism-related genes in *SiR*-impaired lines. Noticeably, enhanced transcripts of the three *APR* genes were quite evident in *SiR*-impaired plants; suggesting that the increased sulfite in the *SiR*-impaired plants could be a result of the reduced *SiR* coupled to enhanced *APR* expression during oxidative stress. Together, our results indicate that *SiR* is involved in oxidative stress tolerance possibly by maintaining sulfite homeostasis, regulating GSH levels, and modulating sulfite metabolism-related gene expression in *Arabidopsis*. *SiR* could be exploited for engineering environmental stress-tolerant plants in molecular breeding of crops.

## Introduction

The assimilatory reduction of inorganic sulfate into organic sulfur compounds proceeds via a highly coordinated mechanism in higher plants (Khan et al., 2010). First, sulfate is adenylated by ATP sulfurylase to adenosine 5-phosphosulfate (APS). Next, APS is reduced to sulfite by the 5-phosphosulfate reductase (APR). The toxic intermediate sulfite is further reduced by sulfite reductase (SiR) to sulfide, which is then incorporated into Cys and other sulfur-containing amino acids and sulfolipids (Leustek and Saito, 1999; Leustek et al., 2000; Nakayama et al., 2000). As an essential enzyme in the sulfate reduction pathway, SiR plays important roles in diverse metabolic processes such as sulfur detoxification and cysteine metabolism (Wirtz et al., 2004; Brychkova et al., 2007; Lang et al., 2007).

Plant SiR is a soluble protein containing one (4Fe-4S) cluster and one siroheme that catalyzes the six-electron reduction of sulfite to sulfide (Lewandowska and Sirko, 2008). *SiR* exists as a single copy in the genome of *Arabidopsis* and is localized exclusively in the plastids (Sekine et al., 2007). SiR acts as a sulfur assimilation enzyme and a chloroplast nucleoid binding protein, indicating that it is essential for the assimilatory sulfate reduction and growth and development of plants (Sekine et al., 2007; Kang et al., 2010; Khan et al., 2010). It has recently demonstrated that SiR plays a role in protecting *Arabidopsis* or tomato plants against sulfite toxicity (Yarmolinsky et al., 2013). Further investigation showed that knockdown expression of SiR resulted in accelerated leaf senescence in tomato plants (Yarmolinsky et al., 2014).

Environmental stresses including abiotic and biotic stress provoke cellular redox imbalances and generate excessive reactive oxygen species (ROS) such as hydroxyl radicals and superoxide ions, which result in oxidative stress in plants (Gechev et al., 2006). When plant cells cannot remove excess ROS promptly, leaves become pale and necrotic. To maintain redox balance and to protect against oxidative stress, plants have evolved a ROS-scavenging system to eliminate excess ROS, including non-enzymatic antioxidants, such as ascorbic acid, glutathione (GSH) and carotenoids, and ROS-removing enzymes. It has been recently shown that impaired-SiR tomato plants significantly decreased GSH levels and led to early leaf senescence (Yarmolinsky et al., 2014). As we know, GSH is both an important reduced sulfur sink and a regulator of sulfur assimilation (Hell, 1997). Also, it plays an important role in protecting plants against oxidative stress (Alscher, 1989; Noctor et al., 1998). However, whether plant SiR participates in oxidative stress response is unclear. In this study, we provide genetic evidence that *SiR* functions in methyl viologen (MV)-induced oxidative stress in *Arabidopsis*.

## Materials and Methods

### Plant Materials and Growth Conditions

*Arabidopsis thaliana* ecotype Columbia (Col-0) was used as the wild type in this study. The wild type and RNAi transgenic seeds were surface sterilized and germinated on plates containing 1/2 Murashige and Skoog (MS) medium. Seeds were stratified at 4°C in darkness for 3 days and then transferred to a growth chamber at 22°C with a 16-h-light/8-h-dark photoperiod. After 1 week, the seedlings were transferred to sterilized low-nutrient soil to obtain fully grown plants. Plants were grown in a growth room at approximately 22°C, 70–80% relative humidity, a photoperiod of 16 h/8 h (day/night) and light intensity of 200 μmol m^-2^ s^-1^, as described before (Xia et al., 2016).

### Real-Time PCR Analysis

Real-time PCR was used to determine expression pattern of *SiR* in different organs and transcript levels of several sulfite network genes (*SiR, SO, SQS1, APR1, APR2*, and *APR3*) in response to MV treatment. Total RNA extraction, first-strand cDNA synthesis and qRT-PCR with gene-specific primers (Supplementary Table S1) were conducted as described previously (Xia et al., 2016). The *Arabidopsis Actin2* transcript was used as an internal control to quantify the relative transcript levels as described (Livak and Schmittgen, 2001; Xia et al., 2016). We had previously compared *Arabidopsis EF-1a, Actin2*, and *Tubulin* as internal controls and found that *Actin2* is more stable than the others as a reference gene in our pilot experiment. All qRT-PCR experiments were performed with three biological and three technical replicates.

### Construction of Plant Expression Vectors and Development of RNAi Transgenic *Arabidopsis* Lines

For the RNA interference (RNAi) construct, a 369-bp-length fragment of *SiR* cDNA was amplified using primers SiR-F and SiR-R (Supplementary Table S1) and introduced as sense and antisense into the binary vector pFGM with *Bam*HI and *Xba*I, or NcoI and SwaI restriction sites, resulting in the transformation construct pFGM-35S:SiR-RNAi.

The binary construct was introduced into *Agrobacterium tumefaciens* (strain GV3101) and then transformed into *Arabidopsis* (Col-0) via the floral dip method (Clough and Bent, 1998). Transformed lines were selected by antibiotic resistance and verified by PCR analysis. Homozygous *Arabidopsis SiR*-modified lines that contained single-site transgene insertions were identified and maintained growth to set seeds until T_3_ generation. Homozygous T3 lines were used for further experiments.

### Analysis of *SiR* RNAi Transgenic *Arabidopsis* Lines for Oxidative Stress Tolerance

The WT and transgenic lines (Ri-1, Ri-4 and Ri-6) were cultured in 1/2 MS medium under a 16 h light/8 h dark cycle at 22°C for 1 week, and then the plants were transplanted into small pots with soil (four plants per pot, and two pots for each line) and grown for 4 weeks under standard growth conditions. Five-week-old WT and RNAi lines were sprayed with 20 μM of MV. Three replicates each consisting of two pots of seedlings from each line were included for both MV treatment and H_2_O-treated controls. The whole experiment was repeated three times. The remaining chlorophyll content of WT and RNAi lines was determined 3 days after treatment. Leaf damage level (LDL) was determined as the ratio of damaged area divided by the whole leaf area. The relative damage level (%) was calculated as the mean of LDL of five leaves from each plant.

### Determination of MDA and H_2_O_2_ Contents

MDA content was determined as described previously (Draper and Hadley, 1990; Huo et al., 2016). H_2_O_2_ content was assayed according to our previously used method (Xia et al., 2012). The absorbance of the resulting solution was measured at 415 nm and the H_2_O_2_ concentration was determined using a standard curve plotted with standard concentrations of H_2_O_2_. In both experiments, three independent biological replicates were conducted, and three times were done in each independent assay.

### Determination of SiR Activity, Sulfite, and Glutathione Contents

Leaf samples were extracted and protein concentration was determined as described previously (Bradford, 1976; Xia et al., 2012). The assay solution contained 10 mM DTT, 25 mM HEPES, pH 7.8, 5 mM OAS, 5 units of OAS-TL, 15 mM Na_2_S_2_O_4_, 30 mM NaHCO_3_, 1 mM Na_2_SO_3_, and 5 mM MV along with the crude leaf extract. SiR activity was determined according to the assay of Khan et al. (2010). Sulfite levels of leaf samples were determined using ion-exchange chromatograph system as described (Brychkova et al., 2012; Xia et al., 2012). GSH was determined according to the method as described by Griffith (1980). For each experiment, three replicates were conducted for each test sample and the experiment was repeated three times.

### Accession Numbers

Sequence data from this article can be found in the GenBank/EMBL data libraries under the following accession numbers: *SiR*, At5g04590; *SO*, At3g01910; *SQS1*, At4g33030; *APR1*, At4g04610; *APR2*, At1g62180; *APR3*, At4g21990.

## Results

### Transcript Profiles of *SiR* in *Arabidopsis* Organs and During Oxidative Stress

**FIGURE 1:**
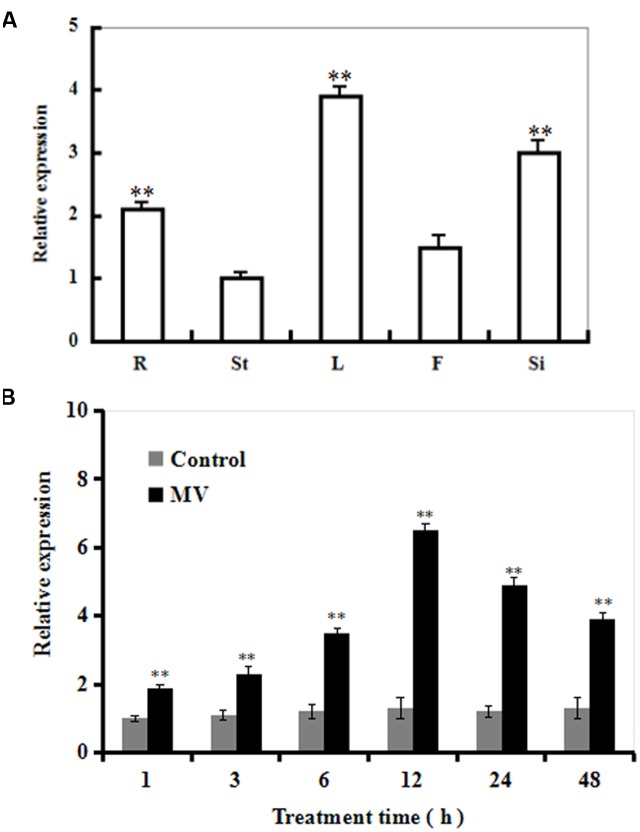
**The transcript profiles of *SiR* in *Arabidopsis* organs and during oxidative stress. (A)** The transcriptional pattern of *SiR* in *Arabidopsis* root (R), stem (St), leaf (L), flowers (F), and immature silique (Si) samples evaluated by qRT-PCR. **(B)** Changes in the *SiR* transcript levels at various time points in response to MV exposure in *Arabidopsis* plants by qRT-PCR. Three-week-old plants were exposed to H_2_O (control) or 10 μM MV and sampled at 1, 3, 6, 12, 24 and 48 h. Leaf samples were collected for qRT-PCR analysis. In both **(A,B)**, *Actin2* was used as an internal control. For each experiment, three technical replicates were conducted. Data shown are Mean ± SE of three independent experiments. ^∗∗^*t*-test, with *P* < 0.01.

The transcriptional pattern of *SiR* was examined in five organs of *Arabidopsis* (roots, stems, leaves, flowers, and immature siliques). The *SiR* transcript levels were significantly high in leaves and immature siliques (**Figure 1A**). In contrast, *SiR* transcripts were low in stems. The relative expression in leaves was three times greater than that in the stems (**Figure 1A**).

Time-course analysis of *SiR* transcript levels in *Arabidopsis* plants in response to oxidative stress was performed by qRT-PCR (**Figure 1B**). The transcript levels of *SiR* were increased rapidly after 1 h, and reached a maximal level at 12 h (about sixfold increase in transcripts) during 48 h period of MV treatment (**Figure 1B**). In contrast, no significant difference was observed under control conditions (**Figure 1B**). These data suggest that *SiR* could be involved in oxidative stress response.

### Generation of *SiR* Knockdown *Arabidopsis* Plants

**FIGURE 2:**
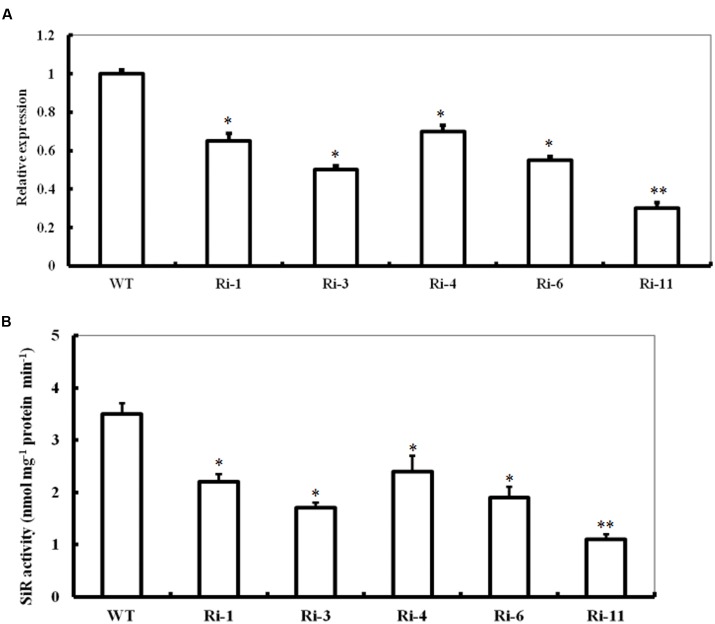
**Expression levels and activity of SiR in *SiR*-modified *Arabidopsis* plants. (A)** Transcript levels of *SiR* in the WT and five RNA interference lines (named Ri-1, Ri-3, Ri-4, Ri-6, and Ri-11) determined by qRT-PCR. **(B)** Total SiR activity in leaf extracts from WT and RNAi lines. In both histograms above, values are mean ± SE. The asterisks indicate significance of the difference from the corresponding control values determined by Student’s *t*-test (^∗∗^*t*-test, with *P* < 0.01; ^∗^*t*-test, with *P* < 0.05).

To further investigate the role of *SiR* in oxidative stress tolerance, transgenic *Arabidopsis* plants under-expressing *SiR* were generated (**Figure 2A**). Due to early seedling lethal caused by SiR inhibition (Khan et al., 2010), only five independent transgenic lines (T1; Ri-1, Ri-3, Ri-4, Ri-6 and Ri-11) were identified by antibiotic-resistance analysis and PCR using specific primers (Supplementary Table S1). qRT-PCR analysis of the T1 generation revealed that these five RNAi lines exhibited 30–70% reductions in SiR transcripts when compared with wild type (**Figure 2A**). Accordingly, these RNAi lines showed significantly reduced levels of SiR activity (30–68% of the wild type level; **Figure 2B**). However, the RNAi line Ri-11 showed severe growth retardation and could not be used for screening homozygous line because of strong suppression in *SiR* expression (**Figures 2A,B**). Among the remaining four lines, three homozygous lines (Ri-1, Ri-4 and Ri-6) had 55–70% of the wild type levels, which were chosen for further experiments.

### Response of *SiR* Under-Expressing *Arabidopsis* Plants to Oxidative Stress

**FIGURE 3:**
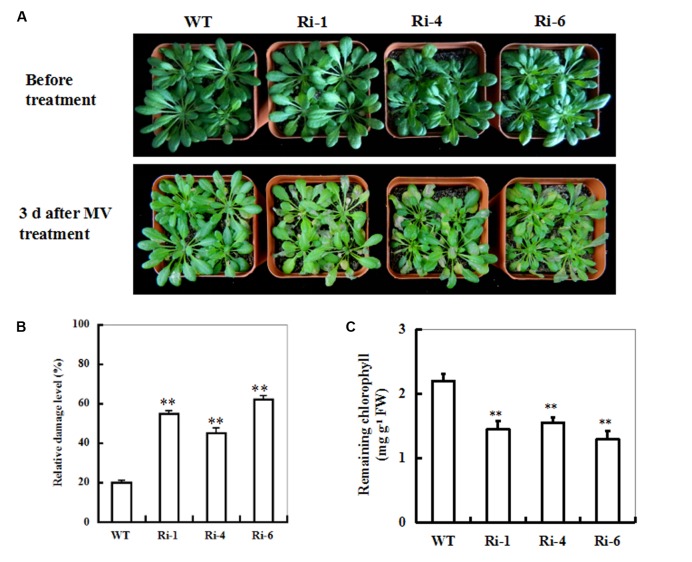
**Responses of wild-type and *SiR* under-expressing plants to oxidative stress. (A)** Toxic effect of MV on WT and *SiR* RNAi plants. Five-week-old WT and RNAi lines (Ri-1, Ri-4 and Ri-6) were sprayed with 20 μM of MV and examined 3 days later. **(B)** Relative damage level in the wild-type and RNAi plants after MV exposure. Values are mean ± SE, *n* = 20. ^∗∗^*t*-test, with *P* < 0.01. **(C)** Residual chlorophyll in the wild-type and RNAi lines after MV exposure. Values are mean ± SE, *n* = 20. ^∗∗^*t*-test, with *P* < 0.01.

To characterize the performance of *SiR* under-expressing *Arabidopsis* under MV-induced oxidative stress, T3 seeds of the three homozygous RNAi lines were directly sown in soil. 20 μM of MV was applied to 5-week-old seedlings by spraying directly onto the leaves. Three days after MV spraying, the RNAi transgenic plants showed relative higher necrosis and wilting than the wild-type plants (**Figure 3A**). Accordingly, the mean LDL of the RNAi lines was about twofold higher than that in the WT (**Figure 3B**) and the remaining chlorophyll content in the RNAi lines was significantly lower than that in WT plants (∼36% on the average; **Figure 3C**). These results demonstrate that impairment of *SiR* in *Arabidopsis* plants decreases tolerance to MV-induced oxidative stress.

### Under-Expression of *SiR* Increases MDA and H_2_O_2_ Accumulations under Oxidative Stress

**FIGURE 4:**
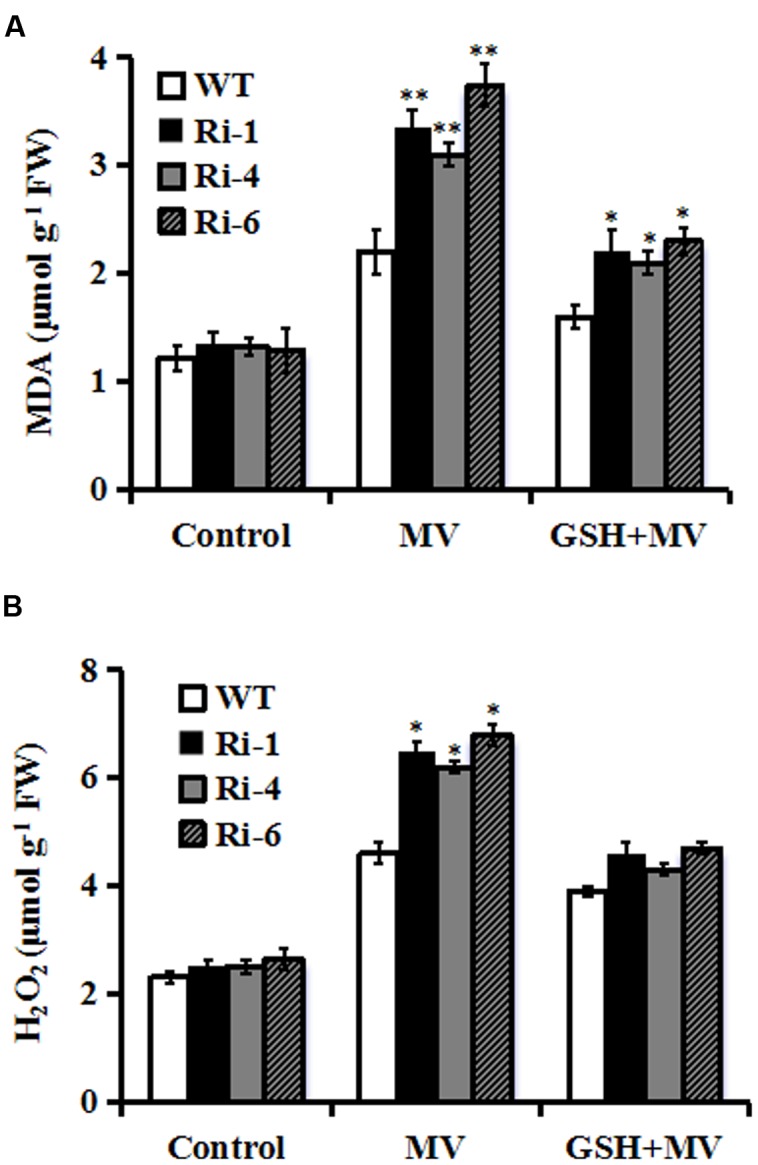
**Changes of MDA and H_2_O_2_ in *SiR* RNAi lines under oxidative stress. (A)** Determination of MDA accumulation in leaves of WT and three RNAi lines 3 days after MV (20 μM) or GSH (10 mg/L) plus MV(20 μM) spraying. **(B)** Quantitative determination of H_2_O_2_ accumulation in leaves of WT and three RNAi lines 3 days after MV (20 μM) or GSH (10 mg/L) plus MV (20 μM) spraying. In both **(A,B)**, each experiment was repeated twice. Bar indicates SE. Values are mean ± SE. ^∗∗^*t*-test, with *P* < 0.01; ^∗^*t*-test, with *P* < 0.05.

Reduced oxidative tolerance in *SiR* RNAi lines prompted us to detect the differences in lipid peroxidation. Malondialdehyde (MDA), a product of lipid peroxidation was measured between the WT and RNAi plants 3 days after MV treatment. The MDA levels in the RNAi lines (149, 133, and 190% increases for RNAi-1, -4, and -6, respectively) were significantly higher than in the WT (only 80% increase), suggesting that the RNAi plants suffered more membrane damage than the wild type (**Figure 4A**). By contrast, in the presence of GSH, the MDA levels in the RNAi lines were only 70% increase on average after MV exposure (**Figure 4A**).

The *SiR* knockdown plants had higher MDA levels under oxidative stress, implying that they may be subjected to more serious oxidative damage than the WT. Therefore, it was of interest to detect ROS accumulation in the WT and RNAi lines during oxidative stress. Quantitative determination of H_2_O_2_ accumulation was performed in 3-day MV-treated leaves along with controls from RNAi and WT plants. As shown in **Figure 4B**, H_2_O_2_ content increased in both WT and transgenic lines after oxidative stress. However, RNAi lines accumulated higher levels of H_2_O_2_ (200% increase averagely) relative to WT (130% increase) after MV treatment (**Figure 4B**). By contrast, in the presence of GSH, there was no significant difference in H_2_O_2_ content between RNAi and WT plants after MV exposure (**Figure 4B**). These physiological indices demonstrated that higher ROS accumulation and lipid peroxidation in the RNAi transgenic lines may be correlated to their decreased tolerance to oxidative stress and was attenuated by GSH.

### Sulfite and Glutathione (GSH) Accumulations in *SiR* Under-Expressing Lines upon MV Exposure

**FIGURE 5:**
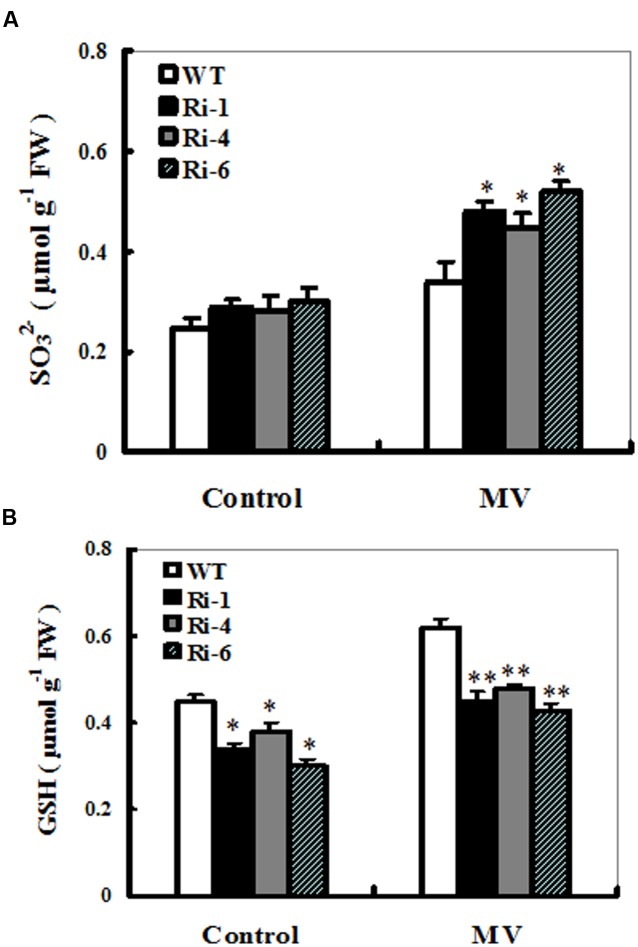
**Sulfite and glutathione accumulations in the wild-type and *SiR* RNAi plants upon MV exposure.** Contents of sulfite **(A)** and glutathione **(B)** were measured 3 days after MV (20 μM) spraying. Each experiment was repeated three times. Bar indicates SE. Values are mean ± SE. ^∗∗^*t*-test, with *P* < 0.01; ^∗^*t*-test, with *P* < 0.05.

To monitor *in planta* changes in the levels of substrate and products upon MV exposure, sulfite and S-metabolite GSH contents were determined in treated and control leaves from wild-type and *SiR* under-expressing plants. The effect of MV on total sulfite levels in these *SiR* under-expressing lines resulted in relatively bigger increases of 61–73% (66, 61, and 73% increases for RNAi-1, -4, and -6, respectively; **Figure 5A**). By contrast, the sulfite content in the leaves of WT plants only increases by 36% upon MV exposure (**Figure 5A**). In addition, under control conditions, sulfite levels in the RNAi lines had no significant increase compared to the wild-type plants (**Figure 5A**).

For changes in the GSH content, a significant increase was detected in the wild-type plants (60% increase), but not in the three RNAi lines (only nearly 30% increase on average; **Figure 5B**). Noticeably, under control conditions, total GSH levels in these RNAi lines had significant reductions compared to the wild-type plants (**Figure 5B**). These results demonstrated that reduced *SiR* expression resulted in excess sulfite accumulation and insufficient biosynthesis of GSH, indicating that *SiR* was involved in MV-induced oxidative stress tolerance largely attributable to GSH levels in plants.

### SiR Knockdown-Triggered Sensitivity to Oxidative Stress Was Reversed by GSH

**FIGURE 6:**
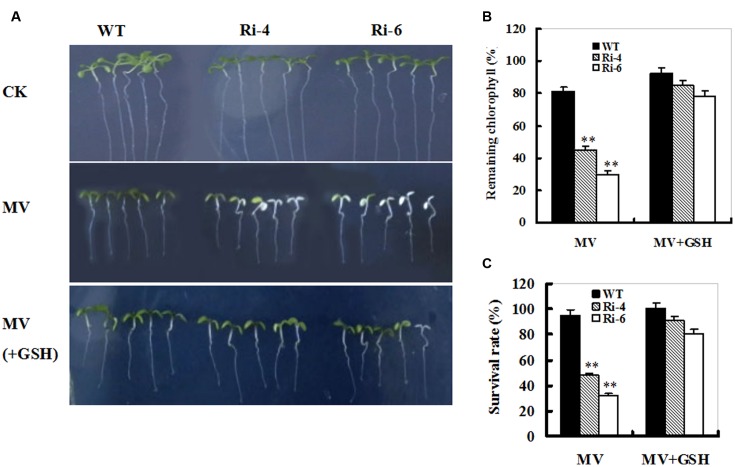
**Effect of exogenous GSH on response of wild-type and *SiR* under-expressing plants to oxidative stress. (A)** Representative growth phenotypes of WT and RNAi seedlings when exposed to MV and MV+GSH. Seven-day-old seedlings of WT and RNAi lines (Ri-4 and -6) were vertically growing on 1/2 MS medium supplemented with 0 (CK), MV(5 μM), and MV(5 μM) plus GSH (10 mg/L) for 7 days. **(B)** Relative residual chlorophyll (%) in the wild-type and RNAi lines after MV and MV plus GSH treatments. Values are mean ± SE, *n* = 15. ^∗∗^*t*-test, with *P* < 0.01. **(C)** Survival rates (%) under MV stress in **(B)** were determined as the number of visibly green plants after 7 days. Values are mean ± SE, *n* = 30. ^∗∗^*t*-test, with *P* < 0.01.

The MV-induced loss of chlorophyll and accelerated cellular damage in *SiR*-impaired plants may largely result from the insufficient GSH levels (**Figures 4** and **5**). To examine this directly, 1-week-old seedlings from WT and *SiR* mutants (Ri-4 and Ri-6) were treated with MV or MV plus GSH for a week. MV exposure caused marked reductions in chlorophyll and survival rates that were alleviated in the presence of GSH in both wild-type and RNAi lines (**Figure 6A**). Interestingly, the WT plants exhibited the lowest chlorophyll degradation and the highest survival of seedlings (**Figures 6B,C**). This observation indicates that GSH may play a protective role in MV-induced oxidative stress.

### Changes in Sulfite Metabolism-Related Gene Expression in WT and *SiR* Under-Expressing Lines upon MV Exposure

**FIGURE 7:**
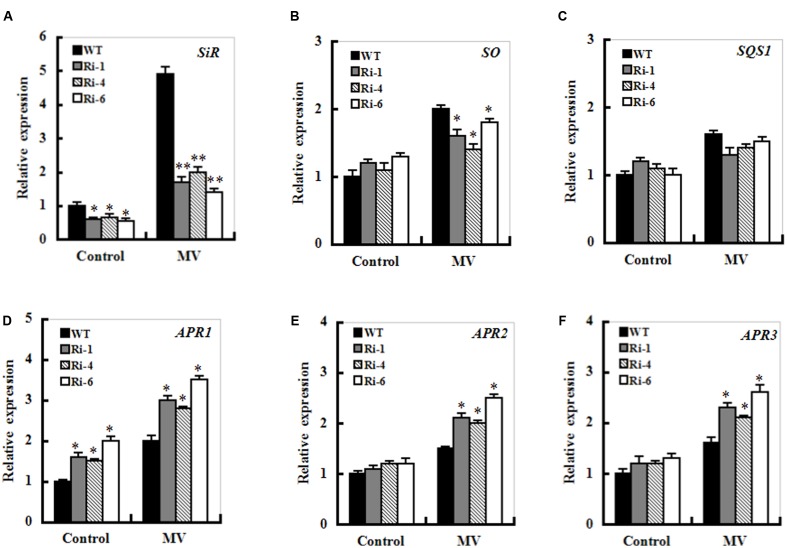
**Effect of *SiR* suppression on transcripts of the sulfite network genes under oxidative stress.** Leaf samples from 5-week-old WT and three RNAi lines were harvested after 12 h of MV (20 μM) spraying and transcriptional expression of *SiR* **(A)**, *SO* **(B)**, *SQD1* **(C)**, *APR1* **(D)**, *APR2* **(E)**, and *APR3* **(F)** was detected by qRT-PCR. mRNA levels of these genes were normalized to the transcripts of *Actin2* in the same samples. For each assay, the expression level of WT under control conditions was taken as 1.0, and data represented mean ± SE of three biological replicates. ^∗∗^*t*-test, with *P* < 0.01; ^∗^*t*-test, with *P* < 0.05.

The transcripts of sulfite metabolism-related enzymes sulfite oxidase (SO), UDP- sulfoquinovose synthase (SQS1), and adenosine-5’-phosphosulfate reductase (APR1, APR2 and APR3) were monitored upon MV exposure in the wild-type and *SiR* under-expression plants by qRT-PCR. After MV exposure for 12 h, the transcripts of all the genes except for the *SQS1* displayed similarly increasing trends between the WT and RNAi plants (**Figure 7**). In particular, enhanced expression of the APR transcripts was quite evident in SiR-impaired plants compared with those in wild-type plants. Moreover, the transcripts of the *APR1* were significantly up-regulated even under normal conditions (**Figures 7D–F**). This suggests that the increased sulfite in the SiR-impaired plants could be a result of the reduced SiR coupled to enhanced APR expression during control and oxidative stress.

## Discussion

As a key enzyme in the sulfate assimilation pathway, SiR is not only involved in plant growth and development, but in stress response (Khan et al., 2010; Yarmolinsky et al., 2013, 2014). Our genetic evidence suggests that SiR participates in oxidative stress tolerance by possibly regulating sulfite homeostasis, GSH levels and sulfite metabolism-related gene expression.

### *SiR* is Evolutionarily Conserved and Functionally Divergent in Plant Species

For the *SiR*, there exists one copy in *Arabidopsis* genome and two copies in the genomes of rice and poplar (Bork et al., 1998; Kopriva, 2006). A phylogenetic tree was established based on SiR protein sequences available in GenBank from 10 plant species including *Arabidopsis*, tobacco, tomato, soybean, castor, rice, barley, wild wheat, sorghum, and maize (**Supplementary Figure S1**). This indicates that *SiR* is evolutionarily conserved in plant species. *SiR* was found to be expressed in nearly all tissue types in *Arabidopsis* (**Figure 1A**), indicating this gene may be constitutively expressed during both vegetative and reproductive growth. Besides leaves, higher transcript levels of the *SiR* were observed in siliques; suggesting that SiR-dependent sulfate assimilation may be involved in sulfur-containing protein biosynthesis during seed development in *Arabidopsis*. Interestingly, relative higher levels of *SiR* transcripts were also found in roots, indicating that *SiR* may play a role in response to nutritional stress such as sulfate deficiency in roots. This notion was supported by the clue that *SiR* mRNA levels were up-regulated when *Arabidopsis* plants were subjected to sulfate starvation (Hell, 1997). Several lines of evidence have recently shown that plant SiR is essential for growth and development (Khan et al., 2010) and participates in sulfite stress response and leaf senescence (Yarmolinsky et al., 2013, 2014); Also, *SiR* was confirmed to be involved in oxidative stress response in *Arabidopsis* (This study). Taken these findings together, it is strongly evidenced that plant *SiR* is functionally divergent.

### SiR-Dependent Sulfite Reduction is Indispensable for Sulfite Homeostasis during Oxidative Stress

SO, SiR, SQS1, and APR are key enzymes that catalyze the diversion of sulfite to other assimilatory pathways (Eilers et al., 2001; Benning, 2007; Brychkova et al., 2013). In this study, transcript levels of the three APR genes (*APR1, APR2, and APR3*) were significantly up-regulated in the *SiR*-impaired lines during MV-induced oxidative stress compared to those in the WT plants (**Figures 7D–F**). Moreover, sulfite content significantly increased in the leaves of the *SiR* knock-down lines (**Figure 5A**). These results suggest that toxic sulfite levels were increasingly accumulated in plant cells during oxidative stress because of enhanced *APR* expression and impaired *SiR*. This observation could be supported by the evidence that *SiR* mutant RNAi plants were severely suffered by SO_2_ stress due to excess sulfite accumulation (Yarmolinsky et al., 2013). Besides SiR-dependent sulfite reduction pathway, sulfite can be incorporated into sulfolipids, which is catalyzed by the chloroplast-localized UDP-sulfoquinovose synthase (SQS1). Noticeably, transcript levels of the *SQS1* were not changed significantly in both WT and *SiR* under-expressing plants upon MV exposure (**Figure 7C**), indicating that sulfolipid synthesis in plastids may not be a predominant pathway for sulfite homeostasis during oxidative stress. Although transcript levels of *SO* were up-regulated upon MV exposure, *SiR-*impaired plants showed more severe damage than the WT. These results demonstrate that *SiR* is indispensable for maintaining sulfite homeostasis during oxidative stress. Further work is needed to dissect the SiR-dependent and SO-dependent sulfite homeostasis networks in *SiR* and *SO* mutants using transcriptome, proteome, or metabolome approaches.

### Glutathione Plays an Important Role in Response to Oxidative Stress for *SiR*-Impaired Plants

Methyl viologen (MV, paraquat) is one of the most widely used herbicides in agriculture. It rapidly enters leaves and then chloroplasts, where it disrupts PSI electron transport, reducing oxygen to ROS. Thus, it can lead to cell toxicity, membrane peroxidation and even cell death. Sulfite is reduced by SiR to form sulfide, which is then incorporated into Cys and other sulfur-containing compounds such as GSH. As a major organic thiol-containing metabolite, GSH has an important role in maintaining redox homeostasis (Nagalakshmi and Prasa, 2001).

In this study, higher hydrogen peroxide and MDA levels, which are hallmarks of oxidative stress were detected upon MV exposure in the *SiR* knock-down transgenic lines (**Figure 4**). Further investigations showed that a significant increase in the GSH content was detected in the WT, but not in the *SiR* under-expressing lines (**Figure 5B**). This indicates that amounts of GSH were influenced by the SiR levels and in return, GSH levels affected oxidative stress response of the *SiR*-impaired plants directly. In addition, the accumulation of hydrogen peroxide in these RNAi lines may also be a consequence of the lower GSH levels, which weakened the antioxidant capacity. In support of this viewpoint, Ding et al. (2009) showed that impairment of glutathione reductase (GR) led to enhanced sensitivity to MV-induced oxidative stress in transgenic tobacco because of the reduced capacity for regeneration of GSH (Ding et al., 2009).

In summary, SiR can protect plants from oxidative stress, and has a potential for application to economically important crop plants. Future work will be needed to dissect the mechanisms in detail by which the *SiR*-regulated sulfate reductive pathway involved in MV-induced oxidative stress response using mutants of sulfite metabolism-related genes in *Arabidopsis*.

## Author Contributions

ZX designed the research. MW, YJ, ZXu, and ZX performed research and conducted data analyses. ZX wrote the manuscript.

## Conflict of Interest Statement

The authors declare that the research was conducted in the absence of any commercial or financial relationships that could be construed as a potential conflict of interest.

## Supplementary Material

FIGURE S1 **Phylogenetic tree based on the amino acid sequence alignment of plant SiRs.** These plant species include *Arabidopsis thaliana* (CAA89154.1), *Solanum lycopersicum* (AFB83709.1), *Ricinus communis* (XP_002513495.1), *Nicotiana benthamiana* (ACN23794.1), *Glycine max* (XP_003540209.1), *Sorghum bicolor* (XP_002441346.1), *Oryza sativa* (NP_001055978.1), *Hordeum vulgare* (BAK03240.1), *Brachypodium distachyon* (XP_003568157.1) and *Zea Mays* (NP_001105302.1). The bootstrap values shown were calculated based on 500 replications. The tree was constructed using the neighbor-joining method.

## References

[B1] Alscher R. G. (1989). Biosynthesis and antioxidant function of glutathione in plants. *Physiol. Plant.* 77 457–464. 10.1111/j.1399-3054.1989.tb05667.x

[B2] Benning C. (2007). Questions remaining in sulfolipid biosynthesis: a historical perspective. *Photosynth. Res.* 92 199–203. 10.1007/s11120-007-9144-6 17334828

[B3] Bork C. Schwenn J. D. Hell R. (1998). Isolation and characterization of a gene for assimilatory sulfite reductase from *Arabidopsis thaliana*. *Gene* 212 147–153. 10.1016/S0378-1119(98)00155-3 9661674

[B4] Bradford M. M. (1976). A rapid and sensitive method for the quantification of microgram quantities of protein utilizing the principal of protein-dye binding. *Anal. Biochem.* 72 248–254. 10.1016/0003-2697(76)90527-3 942051

[B5] Brychkova G. Grishkevich V. Fluhr R. Sagi M. (2013). An essential role for tomato sulfite oxidase and enzymes of the sulfite network in maintaining leaf sulfite homeostasis. *Plant Physiol.* 161 148–164. 10.1104/pp.112.208660 23148079 PMC3532248

[B6] Brychkova G. Xia Z. Yang G. Yesbergenova Z. Zhang Z. Davydov O. (2007). Sulfite oxidase protects plants against sulfur dioxide toxicity. *Plant J.* 50 696–709. 10.1111/j.1365-313X.2007.03080.x 17425719

[B7] Brychkova G. Yarmolinsky D. Fluhr R. Sagi M. (2012). The determination of sulfite levels and its oxidation in plant leaves. *Plant Sci.* 190 123–130. 10.1016/j.plantsci.2012.04.004 22608526

[B8] Clough S. J. Bent A. F. (1998). Floral dip: a simplified method for *Agrobaterium* -mediated transformation of *Arabidopsis thaliana*. *Plant J.* 16 735–743. 10.1046/j.1365-313x.1998.00343.x 10069079

[B9] Ding S. Lu Q. Zhang Y. Yang Z. Wen X. Zhang L. (2009). Enhanced sensitivity to oxidative stress in transgenic tobacco plants with decreased glutathione reductase activity leads to a decrease in ascorbate pool and ascorbate redox state. *Plant Mol. Biol.* 69 577–592. 10.1007/s11103-008-9440-3 19043665

[B10] Draper H. H. Hadley M. (1990). Malondialdehyde determination as index of lipid peroxidation. *Meth. Enzymol.* 86 421–431. 10.1016/0076-6879(90)86135-I 2233309

[B11] Eilers T. Schwarz G. Brinkmann H. Witt C. Richter T. Nieder J. (2001). Identification and biochemical characterization of *Arabidopsis thaliana* sulfite oxidase. A new player in plant sulfur metabolism. *J. Biol. Chem.* 276 46989–46994. 10.1074/jbc.M108078200 11598126

[B12] Gechev T. S. Van Breusegem F. Stone J. M. Denev I. Laloi C. (2006). Reactive oxygen species as signals that modulate plant stress responses and programmed cell death. *Bioessays* 28 1091–1101. 10.1002/bies.20493 17041898

[B13] Griffith O. W. (1980). Determination of glutathione and glutathione disulfide using glutathione reductase and 2-vinylpyridine. *Anal. Biochem.* 106 207–212. 10.1016/0003-2697(80)90139-6 7416462

[B14] Hell R. (1997). Molecular physiology of plant sulfur metabolism. *Planta* 202 138–148. 10.1007/s004250050112 9202491

[B15] Huo Y. Wang M. Wei Y. Xia Z. (2016). Overexpression of the maize psbA gene enhances drought tolerance through regulating antioxidant system, photosynthetic capability, and stress defense gene expression in tobacco. *Front. Plant Sci.* 6:1223 10.3389/fpls.2015.01223 PMC4709446 26793207

[B16] Kang Y. W. Lee J. Y. Jeon Y. Cheong G. W. Kim M. Pai H. S. (2010). In vivo effects of NbSiR silencing on chloroplast development in *Nicotiana benthamiana*. *Plant Mol. Biol.* 72 569–583. 10.1007/s11103-009-9593-8 20047069

[B17] Khan M. S. Haas F. H. Samami A. A. Gholami M. Bauer A. Fellenberg K. (2010). Sulfite reductase defines a newly discovered bottleneck for assimilatory sulfate reduction and is essential for growth and development in *Arabidopsis thaliana*. *Plant Cell* 22 1216–1231. 10.1105/tpc.110.074088 20424176 PMC2879758

[B18] Kopriva S. (2006). Regulation of sulfate assimilation in *Arabidopsis* and beyond. *Ann. Bot.* 97 479–495. 10.1093/aob/mcl006 16464881 PMC2803671

[B19] Lang C. Popko J. Wirtz M. Hell R. Herschbach C. Kreuzwieser J. (2007). Sulphite oxidase as key enzyme for protecting plants against sulphur dioxide. *Plant Cell Environ.* 30 447–455. 10.1111/j.1365-3040.2006.01632.x 17324231

[B20] Leustek T. Martin M. N. Bick J. A. Davies J. P. (2000). Pathways and regulation of sulfur metabolism revealed through molecular and genetic studies. *Annu. Rev. Plant Physiol. Plant Mol. Biol.* 51 141–165. 10.1146/annurev.arplant.51.1.141 15012189

[B21] Leustek T. Saito K. (1999). Sulfate transport and assimilation in plants. *Plant Physiol.* 120 637–644. 10.1104/pp.120.3.637 10398698 PMC1539218

[B22] Lewandowska M. Sirko A. (2008). Recent advances in understanding plant response to sulfur-deficiency stress. *Acta Biochem. Pol.* 55 457–471. 18787711

[B23] Livak K. J. Schmittgen T. D. (2001). Analysis of relative gene expression data using real-time quantitative PCR and the 2^-ΔΔC_T_^ method. *Methods* 25 402–408. 10.1006/meth.2001.1262 11846609

[B24] Nagalakshmi N. Prasa M. N. (2001). Responses of glutathione cycle enzymes and glutathione metabolism to cooper stress in *Scenedesum bijugatns*. *Plant Sci.* 160 291–299. 10.1016/S0168-9452(00)00392-7 11164601

[B25] Nakayama M. Akashi T. Hase T. (2000). Plant sulfite reductase: molecular structure, catalytic function and interaction with ferredoxin. *J. Inorg. Biochem.* 82 27–32. 10.1016/S0162-0134(00)00138-0 11132635

[B26] Noctor G. Arisi A. M. Jouanin L. Kunert K. J. Rennenberg H. Foyer C. H. (1998). Glutathione: biosynthesis, metabolism and relationship to stress tolerance explored in transformed plants. *J. Exp. Bot.* 49 623–647. 10.1093/jexbot/49.321.623

[B27] Sekine K. Fujiwara M. Nakayama M. (2007). DNA binding and partial nucleoid localization of the chloroplast stromal enzyme ferredoxin: sulfite reductase. *FEBS J.* 274 2054–2069. 10.1111/j.1742-4658.2007.05748.x 17371503

[B28] Wirtz M. Droux M. Hell R. (2004). O-acetylserine (thiol) lyase: an enigmatic enzyme of plant cysteine biosynthesis revisited in *Arabidopsis thaliana*. *J. Exp. Bot.* 5 785–1798. 10.1093/jxb/erh201 15258168

[B29] Xia Z. Huo Y. Wei Y. Chen Q. Xu Z. Zhang W. (2016). The *Arabidopsis* LYST INTERACTING PROTEIN 5 acts in regulating abscisic acid signaling and drought response. *Front. Plant Sci.* 7:758 10.3389/fpls.2016.00758 PMC4887465 27313589

[B30] Xia Z. Sun K. Wang M. Wu K. Zhang H. (2012). Overexpression of a maize sulfite oxidase gene in tobacco enhances tolerance to sulfite stress via sulfite oxidation and CAT-mediated H2O2 scavenging. *PLoS ONE* 7:e37383 10.1371/journal.pone.0037383 PMC3365070 22693572

[B31] Yarmolinsky D. Brychkova G. Fluhr R. Sagi M. (2013). Sulfite reductase protects plants against sulfite toxicity. *Plant Physiol.* 161 725–743. 10.1104/pp.112.207712 23221833 PMC3561015

[B32] Yarmolinsky D. Brychkova G. Kurmanbayeva A. Bekturova A. Ventura Y. Khozin-Goldberg I. (2014). Impairment in sulfite reductase leads to early leaf senescence in tomato plants. *Plant Physiol.* 165 1505–1520. 10.1104/pp.114.241356 24987017 PMC4119034

