# Supplementary figures and images for: Impairment of Sulfite Reductase Decreases Oxidative Stress Tolerance in Arabidopsis thaliana
Source: Front Plant Sci. 2016 Dec 2;7:1843. doi: 10.3389/fpls.2016.01843 (PMC5133253; doi:10.3389/fpls.2016.01843)

**Suppl Fig.1**

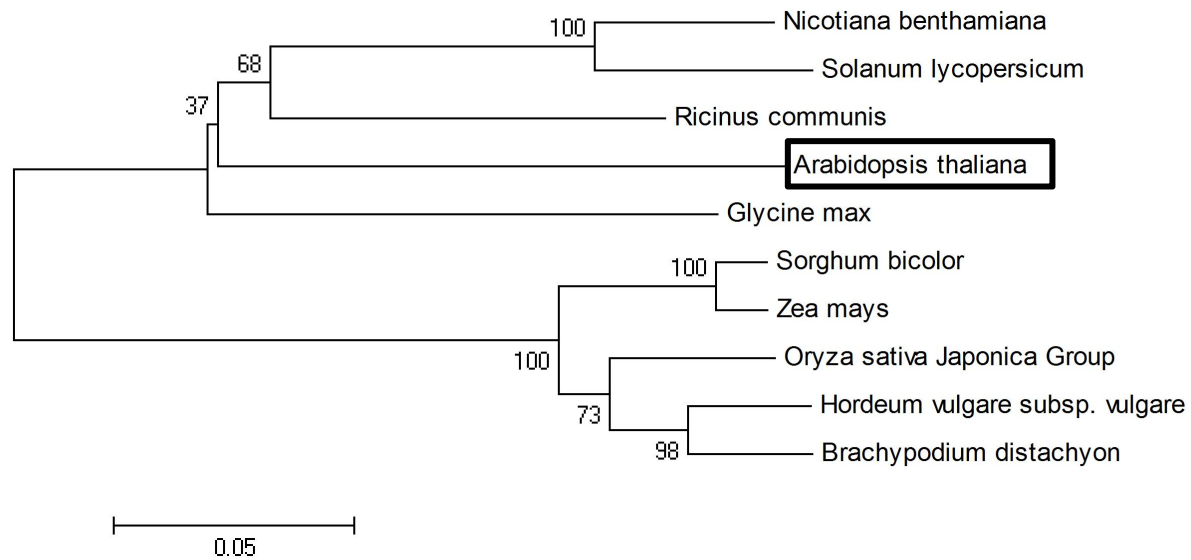

Supplement: FIGURE S1 — Phylogenetic tree based on the amino acid sequence alignment of plant SiRs. These plant species include Arabidopsis thaliana (CAA89154.1), Solanum lycopersicum (AFB83709.1), Ricinus communis (XP_002513495.1), Nicotiana benthamiana (ACN23794.1), Glycine max (XP_003540209.1), Sorghum bicolor (XP_002441346.1), Oryza sativa (NP_001055978.1), Hordeum vulgare (BAK03240.1), Brachypodium distachyon (XP_003568157.1) and Zea Mays (NP_001105302.1). The bootstrap values shown were calculated based on 500 replications. The tree was constructed using the neighbor-joining method. [file Image_1.pdf]
