# Supplementary material for: Impairment of Sulfite Reductase Decreases Oxidative Stress Tolerance in Arabidopsis thaliana
Source: Front Plant Sci. 2016 Dec 2;7:1843. doi: 10.3389/fpls.2016.01843 (PMC5133253; doi:10.3389/fpls.2016.01843)
Supplement: Supplementary file 2 [file Table_1.pdf]

Table S1 Primers used in this study.

Primer name	Primer sequence*	Use
SiR-QF	5'-TTTGAAAAGGTTGGTCTGGACTACG-3'	qRT-PCR for *SiR*
SiR-QR	5'-GCTATCTGGGTCAGGTTCGGTGT-3'	expression
SO-QF	5'-AAAGACATCAGGTCCCTCCCAAAGTA-3'	qRT-PCR for *SO*
SO-QR	5'-CAATAGCAGAAACATCCCATCCAAC-3'	expression
SQS1-QF	5'-TATGGTAAAGGTGGTCAGACGAGAGG-3'	qRT-PCR for *SQS1*
SQS1-QR	5'-GTCATCTTTTTCACGTCTAGCCCAAG-3'	expression
APR1-QF	5'-CTCGTTTCGGTGTTTCATTG-3'	qRT-PCR for *APR1*
APR1-QR	5'-CAATCCCTTGCTCCTAACCA-3'	expression
APR2-QF	5'- CCACACATCAGCTCCTTCAA-3'	qRT-PCR for *APR2*
APR2-QR	5'-AACGCTGAGTCACATTCACG-3'	expression
APR3-QF	5'-TCCAAGCACGTAAACCCTTC-3'	qRT-PCR for *APR3*
APR3-QR	5'-CGGCTTCTCTGAGTTTGTCC-3'	expression
SiR-F	5′- ACTCTAGACCATGGCTTCGTCGAGTTCTTCGCCTAT-3′	Construction of *SiR*
SiR-R	5′- ACGGATCCATTTAAATTGAAACGTCTGCCTTGTGGT-3′	under-expression
		vector
Actin2-F	5'-TTGTGCTGGATTCTGGTGATGG-3'	qRT-PCR for
Actin2-R	5'-CCGCTCTGCTGTTGTGGTG-3'	*Actin2* expression

*The underlined nucleotides constitute *Bam*HI (GGATCC), *Nco*I (CCATGG), *Swa*I (ATTTAAAT), or *Xba*I (TCTAGA) restriction enzyme digestion sites.
